# Neurotoxocarosis: marked preference of *Toxocara canis* for the cerebrum and *T. cati* for the cerebellum in the paratenic model host mouse

**DOI:** 10.1186/1756-3305-7-194

**Published:** 2014-04-22

**Authors:** Elisabeth Janecek, Andreas Beineke, Thomas Schnieder, Christina Strube

**Affiliations:** 1 Institute for Parasitology, University of Veterinary Medicine Hannover, Buenteweg 17, 30559 Hannover, Germany; 2 Department of Pathology, University of Veterinary Medicine Hannover, Buenteweg 17, 30559 Hannover, Germany

**Keywords:** *Toxocara canis*, *Toxocara cati*, Roundworms, Larval migration, Neurotoxocarosis, Zoonotic helminths

## Abstract

**Background:**

Infective larvae of the worldwide occurring zoonotic roundworm *T. canis* exhibit a marked affinity to the nervous tissues of paratenic hosts. In humans, most cases of neurotoxocarosis are considered to be caused by larvae of *T. canis* as *T. cati* larvae have rarely been found in the CNS in previous studies. However, direct comparison of studies is difficult as larval migration depends on a variety of factors including mouse strains and inoculation doses. Therefore, the present study aims to provide a direct comparison of both roundworm species in mice as a model for paratenic hosts with specific focus on the CNS during the acute and chronic phase of disease to provide a basis for further studies dealing with neurotoxocarosis.

**Methods:**

C57Bl/6J mice were infected with 2000 embryonated *T. canis* and *T. cati* eggs, respectively as well as Balb/c mice infected with *T. cati* eggs only. On 8 time points post infection, organs were removed and microscopically examined for respective larvae. Special focus was put on the CNS, including analysis of larval distribution in the cerebrum and cerebellum, right and left hemisphere as well as eyes and spinal cord. Additionally, brains of all infection groups as well as uninfected controls were examined histopathologically to characterize neurostructural damage.

**Results:**

Significant differences in larval distribution were observed between and within the infection groups during the course of infection. As expected, significantly higher recovery rates of *T. canis* than *T. cati* larvae were determined in the brain. Surprisingly, significantly more *T. canis* larvae could be found in cerebra of infected mice whereas *T. cati* larvae were mainly located in the cerebellum. Structural damage in brain tissue could be observed in all infection groups, being more severe in brains of *T. canis* infected mice.

**Conclusions:**

The data obtained provides an extensive characterization of migrational routes of *T. canis* and *T. cati* in the paratenic host mouse in direct comparison. Even though to a lesser extent, structural damage in the brain was also caused by *T. cati* larvae and therefore, the potential as pathogenic agents should not be underestimated.

## Background

*Toxocara* spp. are worldwide occurring helminths of carnivores with high zoonotic potential [1]. Larvae undergo a full development into the adult stage in the definitive host “carnivore” which sheds large amounts of eggs into the environment. Under suitable environmental conditions, the infective third stage larva develops in the egg [2,3], which may be taken up by paratenic hosts, including humans. After ingestion, larvae hatch in the small intestine, migrate through the paratenic host’s organs and persist as the infectious stadium in different tissues for prolonged periods of time. Additionally, consumption of infected paratenic hosts may result in infection as persisting larvae are reactivated and continue migration [4-8]. Migrational behaviour and structural damage caused by larvae of the canine roundworm *T. canis* have been characterized extensively in several paratenic hosts finding a strong neural affinity whereas migrational behaviour and damages caused by *T. cati* larvae have been neglected [9-11]. Even though both roundworm species share antigenic fractions and behave similarly in animal models after hatching, the zoonotic potential of *T. cati* is often underestimated [11]. It is assumed, that most human cases of neurotoxocarosis result from infection with *T. canis* as no reliable differentiation between *T. canis* and *T. cati* larvae is established and previous studies mainly revealed accumulation of *T. canis* larvae in brains of infected mice [9,12-14]. In contrast, few studies report accumulation of *T. cati* larvae mainly in the muscle and only low larval numbers in the brain [15-18].

Human neurotoxocarosis assumingly caused by *T. canis* larvae has been described in several cases; however, occurrence is rather rare. Commonly described clinical symptoms in humans have been behavioral disorders, focal or generalized seizures, ataxia, sensory disturbances, brain infarcts as well as urinary retention [19-23]. Sporadically, cognitive dysfunctions and reduced ability of long and short term memory have been reported [20,22,24]. Infrequently, *T. cati* infection has been suggested with the patient presenting symptoms like mild spastic paraplegia, superficial sensory impairment, urinary retention and slightly brisk deep tendon reflexes [25].

Mice are a valuable model for characterization of infection progress as they present similar clinical manifestations compared to humans. Behavioural changes like abnormal social behaviour, increase of immobility, decrease of exploratory behaviour as well as impairment of learning and memory have been observed in mice infected with *T. canis*[26-29]. Furthermore, central nervous symptoms such as dullness, paresis and tremor were described accompanied by pathological findings like demyelination, focal malacia and mixed cell infiltration [27,30]. As migrational behaviour differs between mouse strains and infection doses [30,31], direct comparison of both roundworm species under equal experimental parameters is essential. The chronic course of a *T. cati* infection in comparison to *T. canis* infection focusing on the CNS has not been clearly characterized yet. Therefore, the aim of the present study was to directly compare migration routes of *T. canis* and *T. cati* in C57Bl/6J (B6) mice as well as *T. cati* in Balb/c mice and observe brain tropism for both species as well as possible neuropathological changes resulting from migration to the brain. This provides a valuable model to extrapolate results to human neurotoxocarosis and serves as a foundation for further molecular studies.

## Methods

### Animal models and infectious material

Animal experiments were permitted by the ethics commission of the Lower Saxony State Office for Consumer Protection and Food Safety under reference numbers 33.14-42502-04-11/0336 and 33.9-42502-05-01A038. Mouse strains C57Bl/6JRccHsd mice as well as Balb/cOlaHsd mice (Harlan Laboratories, Horst, Netherlands) were used as model organisms. Infective material consisted of *T. canis* and *T. cati* eggs obtained from feces of experimentally infected dogs and cats, respectively. Eggs were purified by combined sedimentation- flotation method with subsequent rinsing in tap water. Eggs were incubated at 25°C for 4 weeks for embryonation.

Mice were infected orally with 2000 embryonated *T. canis* or *T. cati* eggs, respectively in a total volume of 0.5 ml tap water at five weeks of age. Infection groups were divided into 64 (32 male/32 female) Balb/c mice infected with *T. cati,* 64 (32 male/32 female) C57Bl/6J (B6) mice infected with *T. cati* and 64 (32 male/32 female) B6 mice infected with *T. canis*. As the literature provides extensive data concerning *T. canis* migrational routes in Balb/c mice, this strain was omitted for *T. canis* as sufficient data is available for comparison. A placebo group of 16 B6 mice was given 0.5 ml tab water. At day 2, 7, 14, 21, 28, 35, 42 and 98 post infection (pi), eight mice per infection group as well as two control mice were euthanized by cervical dislocation. Lungs, liver, heart, kidneys, spleen, muscle, eyes, spinal cord and the brain were removed and stored individually at −80°C until digestion. Out of the eight brains per group, two were used for subsequent histopathological examination and six brains were divided into cerebrum, cerebellum and subdivided into left and right hemisphere before freezing at −80°C.

### Pathological examination

Organs were examined macroscopically at dissections. For histopathological examination, two brains (one male and one female mouse) of each group were fixed in 10% formalin for at least three days. Brains were cut coronally at the bregma zero coordinate as well as about 5 mm to the left and 3 and 7 mm to the right of the bregma zero coordinate according to the mouse brain atlas [32] and embedded in paraffin. Embedded sections were cut to obtain 2 μm thick slices, which were stained with haematoxylin and eosin (H & E stain).

### Artificial digestion

Determination of the larval counts in the organs was accomplished by artificial digestion and subsequent microscopical count. Organs were thawed and cut into small pieces which were digested in a solution containing 300 mM HCl (Carl Roth GmbH, Karlsruhe, Germany) and 1% pepsin (Merck, Darmstadt, Germany). Incubation occurred at 37°C in a water bath while constantly stirring for approximately 2 hours. Solution was centrifuged at 3000 × *g* for 30 min. The supernatant was discarded and the pellet microscopically examined for larval counts.

### Statistical analysis

Statistical analyses were carried out using SAS software (version 9.3). Analysis of single organs over time and between groups was conducted using 2-way ANOVA with subsequent Ryan-Einot-Gabriel-Welsch multiple range test to control experimentwise error rate. Comparisons of larval distribution between individual organs were made using least significant difference (LSD) post hoc tests.

## Results

### General larval distribution

Artificial digestion revealed statistically significant differences between *T. canis* and *T. cati* larval migration. On day 2 pi, significantly more *T. canis* than *T. cati* larvae were detected in the liver. In contrast, significantly less *T. canis* larvae could be detected in lungs at this time point- with significantly more *T. cati* larvae in lungs of B6 than in Balb/c mice. Looking at the course of infection within infection groups, larval counts in liver and lungs decreased significantly after day 2 in Balb/c *T. cati* infected mice. Larval counts in lungs and livers of B6 *T. cati* and in livers of *T. canis* infected mice decreased significantly starting day 2 pi. Larval counts in liver and lungs of all infection groups remained on a low level during the later time points. During all time points post infection, significantly more *T. cati* than *T. canis* larvae could be recovered from the muscle whereas no significant differences between muscle larvae recovery rates were observed for Balb/c and B6 *T. cati* infected mice.

In the heart, kidneys and spleen, larvae could be recovered only sporadically and in very low numbers, rarely exceeding one larva on average, in all infection groups during the entire study. Therefore, they were excluded from statistical analysis.

**Figure 1:**
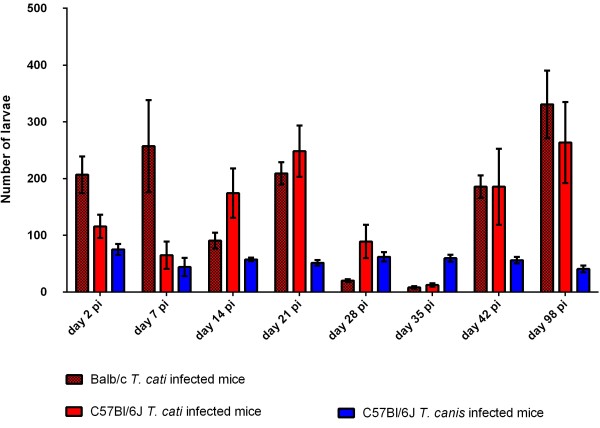
**Total recovered larvae.** Total mean number of *T. cati* and *T. canis* larvae recovered from experimentally infected Balb/c and B6 mice during different time points post infection. Error bars indicate standard errors of the mean (SEM).

A detailed overview of larval numbers as well as recovery rates in the examined organs is given in Table 1. Overall, the total number of recovered larvae was much higher in *T. cati* infected mice whereas only very few *T. canis* larvae could be recovered based on the total infection dose. Larval recovery rates for all infection groups during the course of infection are graphically shown in Figure 1. Detailed data concerning statistically significant differences between and within infection groups over the course of infection are shown in Tables 2 and 3.

**Table 1:** Larval distribution and recovery rates

**Organ**	**Infection group**	**Day pi**							
		**2**	**7**	**14**	**21**	**28**	**35**	**42**	**98**
**Liver**	*T. cati* Balb/c	53.7	3.1	0.9	1.3	0.1	0.0	0.6	2.2
		[24.53]	[1.08]	[1.56]	[0.57]	[0.83]	[0.00]	[0.53]	[0.98]
	*T. cati* B6	27.0	0.9	1.6	1.3	0.0	0.0	0.4	0.8
		[21.19]	[1.33]	[1.90]	[0.47]	[0.00]	[0.00]	[0.32]	[0.35]
	*T. canis* B6	65.8	2.6	1.5	1.5	0.9	1.5	1.1	1.3
		[89.3]	[10.28]	[2.69]	[3.33]	[1.52]	[3.33]	[2.10]	[3.52]
**Lung**	*T. cati* Balb/c	80	1.3	0.6	0.3	0.0	0.0	0.3	0.6
		[35.97]	[0.35]	[0.5]	[0.11]	[0.00]	[0.00]	[0.16]	[0.29]
	*T. cati* B6	49.9	15.3	0.3	5.3	0.8	0.3	0.4	0.4
		[47.38]	[22.51]	[0.14]	[4.07]	[0.42]	[1.79]	[0.46]	[0.20]
	*T. canis* B6	5.6	2.4	0.3	0.4	1.0	2.3	0.8	0.1
		[9.10]	[9.87]	[0.40]	[0.81]	[1.54]	[4.31]	[1.27]	[0.37]
**Muscle**	*T. cati* Balb/c	60.4	246.7	83.9	203.8	20.0	7.6	172.0	313.8
		[32.23]	[85.39]	[91.62]	[97.53]	[98.33]	[96.48]	[92.53]	[95.18]
	*T. cati* B6	33.0	45.4	169.9	235.6	87.5	11.4	179.6	255.3
		[26.93]	[69.55]	[96.89]	[92.61]	[97.68]	[91.95]	[97.23]	[95.89]
	*T. canis* B6	0.4	14.3	42.4	37.0	50.4	34.0	26.4	26.8
		[0.49]	[39.85]	[74.54]	[72.88]	[82.83]	[59.86]	[51.95]	[58.27]
**CNS including eyes**	*T. cati* Balb/c	6.8	6.2	6.1	4.3	0.2	0.7	15.3	22.9
		[3.65]	[17.14]	[6.36]	[1.79]	[1.11]	[4.70]	[8.50]	[5.48]
	*T. cati* B6	4.0	3.8	3.8	7.4	1.1	0.3	6.8	9.4
		[2.59]	[7.77]	[1.48]	[3.41]	[2.52]	[2.78]	[2.95]	[4.65]
	*T. canis* B6	0.3	33.9	17.4	16.2	12.5	28.7	36.3	22.9
		[0.31]	[47.43]	[29.28]	[29.22]	[17.35]	[42.79]	[57.50]	[48.96]
**Total number of larvae**	*T. cati* Balb/c	206.7	257.1	90.6	209.1	20.3	8.1	185.6	330.8
		[10.34]	[12.86]	[4.53]	[10.46]	[1.02]	[0.41]	[9.28]	[16.54]
	*T. cati* B6	115.8	65.0	174.4	248.3	89.0	12.0	185.6	263.5
		[5.79]	[3.25]	[8.72]	[12.42]	[4.45]	[0.60]	[9.28]	[13.18]
	*T. canis* B6	75.1	44.1	57.4	51.4	62.0	59.8	56.1	40.6
		[3.76]	[2.21]	[2.87]	[2.57]	[3.1]	[2.99]	[2.81]	[2.03]

Mean larval numbers as well as recovery rates in [%] for examined organs of *T. cati* and *T. canis* infected mice. The recovery rate is based on the total number of larvae found in infected animals.

**Table 2:** Statistically significant differences of organ larval distribution between infection groups

	**Day pi**							
	**2**	**7**	**14**	**21**	**28**	**35**	**42**	**98**
***T. cati *** **Balb/c vs. *T. cati *** **B6**	Lu							Cer, LH
***T. cati *** **Balb/c vs. *T. canis *** **B6**	Li, Lu, Mu	Br, Mu	Br, Ceb, LH, RH, Mu	Br, Ceb, LH, RH, Li, Mu	Br, Ceb, Cbell, LH, RH, Mu	Ceb, Cbell, LH, RH, Mu	Br, Ceb, LH, Lu, Mu	Br, Ceb, Cbell, Mu
***T. cati *** **B6 vs. *T. canis *** **B6**	Li, Lu, Mu	Br, Mu	Br, Ceb, LH, RH, Mu	Br, Ceb, LH, RH, Li, Mu	Br, Ceb, Cbell, LH, RH, Mu	Ceb, Cbell, LH, RH, Mu	Br, Ceb, LH, RH, Lu, Mu	Br, Ceb, LH, Mu

Larval distribution in single organs differing significantly between infection groups at a given day pi. Significances were calculated using 2 –way ANOVA.

Br = total brain, Ceb = cerebrum, Cbell = cerebellum, LH = left hemisphere, RH = right hemisphere, Li = liver, Lu = lungs, Mu = muscle.

**Table 3:** Statistically significant differences of organ larval distribution within infection groups over course of infection

		**Day pi**							
**Day pi**	**Infection group**	**2**	**7**	**14**	**21**	**28**	**35**	**42**	**98**
**2**	*T. cati* Balb/c	-	Ceb, Li, Lu, Mu	Ceb, Li, Lu, Mu	Ceb, Li, Lu, Mu	Ceb, Li, Lu, Mu	Ceb, Li, Lu, Mu	Ceb, Cbell, RH, Li, Lu, Mu	Cbell, LH, RH, Li, Lu, Mu
	*T. cati* B6	-	Li, Lu, Mu	Li, Lu, Mu	Li, Lu, Mu	Li, Lu, Mu	Li, Lu, Mu	Li, Lu, Mu	Li, Lu, Mu
	*T. canis* B6	-	Br, Ceb, Li, Mu	Br, Li, Mu	Br, Li, Mu	Li, Mu	Br, Cbell, Li, Mu	Br, Cbell, LH, Li, Mu	Br, Li, Mu
**7**	*T. cati* Balb/c	Li, Lu, Mu	-					Cbell, RH	Cbell, LH, RH
	*T. cati* B6	Li, Lu, Mu	-	Lu, Mu	Lu, Mu	Lu, Mu	Lu, Mu	Lu, Mu	Lu, Mu
	*T. canis* B6	Br, Ceb, Li, Mu	-	Mu	Mu	Br, Mu			
**14**	*T. cati* Balb/c	Li, Lu, Mu		-				Cbell, RH	Cbell, LH, RH
	*T. cati* B6	Li, Lu, Mu	Lu, Mu	-					
	*T. canis* B6	Br, Li, Mu	Mu	-				Br	
**21**	*T. cati* Balb/c	Li, Lu, Mu			-			Cbell, RH	Cbell, LH, RH
	*T. cati* B6	Li, Lu, Mu	Lu, Mu		-				
	*T. canis* B6	Br, Li, Mu	Mu		-			Br, Cbell	
**28**	*T. cati* Balb/c	Li, Lu, Mu				-		Cbell, RH	Cbell, LH, RH
	*T. cati* B6	Li, Lu, Mu	Lu, Mu			-			
	*T. canis* B6	Li, Mu	Br, Mu			-	Br	Br, Mu	Br
**35**	*T. cati* Balb/c	Li, Lu, Mu					-	Cbell, RH	Cbell, LH, RH
	*T. cati* B6	Li, Lu, Mu	Lu, Mu				-	Mu	
	*T. canis* B6	Br, Cbell, Li, Mu				Br, Cbell	-		
**42**	*T. cati* Balb/c	Cbell, RH, Li, Lu, Mu	Cbell, RH	Cbell, RH	Cbell, RH	Cbell, RH	Cbell, RH	-	Cbell, LH
	*T. cati* B6	Li, Lu, Mu	Lu, Mu					-	
	*T. canis* B6	Br, Cbell, LH, Li, Mu		Br	Br, Cbell	Br, Cbell, Mu		-	
**98**	*T. cati* Balb/c	Cbell, LH, RH, Li, Lu, Mu	Cbell, LH, RH	Cbell, LH, RH	Cbell, LH, RH	Cbell, LH, RH	Cbell, LH, RH	Cbell	-
	*T. cati* B6	Li, Lu, Mu	Lu, Mu						-
	*T. canis* B6	Br, Li, Mu				Br			-

Larval distribution in single organs within infection groups over the course of infection. Respective comparisons of days pi for each infection group are displayed. Significances were calculated using 2 –way ANOVA.

*Br* = total brain, *Ceb* = cerebrum, *Cbell* = cerebellum, *LH* = left hemisphere, *RH* = right hemisphere, *Li* = liver, *Lu* = lungs, *Mu* = muscle.

### Larval distribution in the CNS

Larval distribution in brains revealed statistically significant differences between *T. canis* and *T. cati* infected mice whereas significantly more larval numbers were detected in brains of *T. canis* infected mice with the exception of day 2 pi (cf. Table 2). Within *T. cati* infection groups, significantly lower larval numbers were recovered from brain compared to muscle tissue for each time point post infection. Detailed data concerning comparison of organs and brain within infection groups including corresponding p-values are provided in Table 4.

**Table 4:** P-values resulting from statistical comparison of larval distribution between organs within infection groups

	**Infection group**	**Day pi**							
		**2**	**7**	**14**	**21**	**28**	**35**	**42**	**98**
**Brain vs. lungs**	*T. cati* Balb/c	0.0550	0.3699	0.0323*	0.0209*	0.3632	0.1782	<.0001*	0.0036*
	*T. cati* B6	0.0008*	0.3945	0.0749	0.4868	0.2467	0.9724	0.0012*	0.0001*
	*T. canis* B6	0.0434*	0.0179*	<.0001*	<.0001*	0.0107*	0.0015*	<.0001*	<.0001*
**Brain vs. liver**	*T. cati* Balb/c	0.3669	0.5658	0.1847	0.0891	n.a.	0.1782	0.0008*	0.0082*
	*T. cati* B6	0.0001*	0.0520	0.7520	0.0039*	0.2179	0.3632	0.0344*	<.0001*
	*T. canis* B6	<.0001*	0.0271*	0.0007*	0.0004*	0.0211*	<.0001*	<.0001*	<.0001*
**Brain vs. muscle**	*T. cati* Balb/c	0.009*	0.0333*	0.001*	<.0001*	<.0001*	0.0015*	0.0002*	0.0013*
	*T. cati* B6	0.0002*	0.0020*	<.0001*	0.0002*	0.0002*	0.0005*	<.0001*	<.0001*
	*T. canis* B6	0.2754	0.7050	0.0017*	0.0016*	0.0119*	0.5447	0.3359	0.7466
**Cerebrum vs. cerebellum**	*T. cati* Balb/c	0.4956	0.0264*	0.0355*	0.7864	0.3632	n.a.	0.0403*	0.0903
	*T. cati* B6	0.4063	0.0454*	0.0705	0.1195	0.6109	0.3632	0.8607	0.0033*
	*T. canis* B6	1.0000	0.0154*	0.2918	0.0040*	0.0003*	0.0465*	0.0441*	0.0136*
**Left vs. right hemisphere**	*T. cati* Balb/c	0.3610	0.2636	0.5224	0.9766	0.3632	1.0000	0.1424	0.7825
	*T. cati* B6	0.4063	0.9585	0.1787	0.9075	0.6109	n.a.	0.2565	0.4993
	*T. canis* B6	0.1747	0.7284	0.1622	0.3854	0.6336	0.7935	0.4490	0.9738

Comparison of larval distribution between single organs at a given day pi for each infection group. P-values were calculated using LSD post hoc tests.

*: statistically significant (p ≤ 0.05).

n.a.: not applicable (too low larvae numbers for reliable comparison).

**Figure 2:**
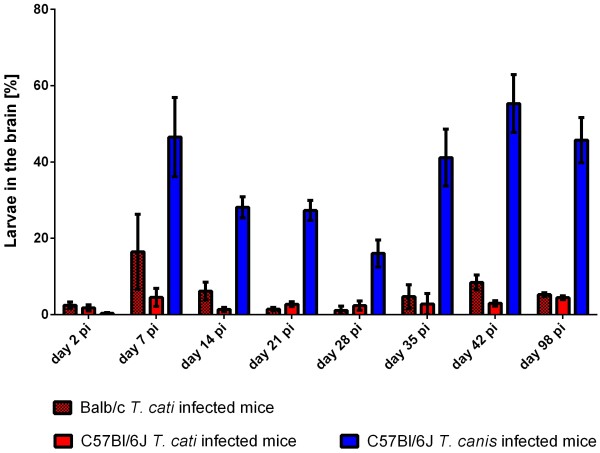
**Larval recovery rates in the brain.** Recovery rates in [%] of *T. cati* and *T. canis* larvae from the brain based on the total number of recovered larvae in experimentally infected mice during different time points pi. Error bars indicate standard errors of the mean (SEM).

**Figure 3:**
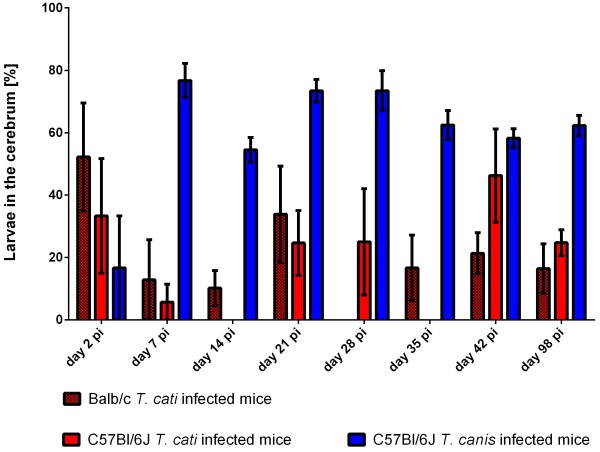
**Larval recovery rates in the cerebrum.** Recovery rates in [%] of *T. cati* and *T. canis* larvae from the cerebrum based on the total larval number found in the brain of experimentally infected mice during different time points pi. Error bars indicate standard errors of the mean (SEM).

**Figure 4:**
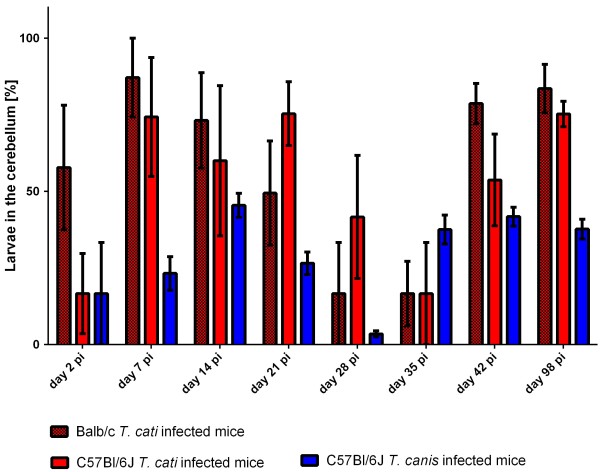
**Larval recovery rates in the cerebellum.** Recovery rates in [%] of *T. cati* and *T. canis* larvae from the cerebellum based on the total larval number found in the brain of experimentally infected mice during different time points pi. Error bars indicate standard errors of the mean (SEM).

On day 2 pi, the brain recovery rate (Figure 2) for all infection groups was low resulting on average in 2.4% and 1.8% in Balb/c and B6 *T. cati* infected mice as well as 0.3% in B6 *T. canis* infected mice. On day 7 pi, the recovery rate of *T. cati* larvae in Balb/c mice was rather high with 16.5% when compared to B6 *T. cati* infected mice (4.5%); however, no significant difference was observed. In contrast, 46.6% of recovered *T. canis* larvae were found in the brain at day 7 pi. In Balb/c as well as B6 *T. cati* infected mice, recovery rates in the brain remained rather low during the remaining course of infection (4.5% on average in Balb/c mice and 2.73% on average in B6 *T. cati* infected mice). In Balb/c *T. cati* infected mice, recovery rates differed significantly on day 7 pi vs. day 21 and 28 pi, at which larval counts dropped to a minimum. No significant differences during the course of infection were found in B6 *T. cati* infected mice. *T. canis* infected mice revealed a rather high recovery rate in the brain with an average of 32.57%. Significant differences were observed on day 2 pi vs. the succeeding time points, as well as day 7 pi vs. day 28 and 42 pi, respectively. Larval distribution in the cerebrum compared to the cerebellum revealed significant differences between *T. cati* and *T. canis* infected mice with statistically significantly higher larval recovery rates in the cerebra of *T. canis* than in *T. cati* infected mice beginning from day 14 throughout day 98 pi. In contrast, significantly higher recovery rates were observed in cerebella of *T. cati* infected mice on day 28, 35 and 98 pi when compared to *T. canis* infected mice. Between both *T. cati* infection groups, no statistically significant differences were observed. Larval recovery rates from the cerebellum and cerebrum are graphically displayed in Figures 3 and 4. Concerning larval distribution in the right and left hemisphere, no significant differences were found between left and right parts of the brain within the infection groups at the different time points post infection. Significant differences between infection groups over the course of infection for cerebellum and cerebrum as well as right and left hemisphere are provided in Table 2.

Analysis of eyes and spinal cords revealed no differences between the infection groups. Only few larvae could be recovered from these organs (cf. Table 5) with larval counts ranging between 0.0 and 1.6 larvae on average during the entire course of infection. Therefore, eyes and spinal cord were excluded from subsequent statistical analysis.

**Table 5:** Larval distribution and recovery rates in CNS organs

**CNS organ**	**Infection group**	**Day pi**																							
		**2**			**7**			**14**			**21**			**28**			**35**			**42**			**98**		
		**LH**	**RH**	**T**	**LH**	**RH**	**T**	**LH**	**RH**	**T**	**LH**	**RH**	**T**	**LH**	**RH**	**T**	**LH**	**RH**	**T**	**LH**	**RH**	**T**	**LH**	**RH**	**T**
Cerebrum	*T. cati* Balb/c	1.4	1.2	**2.6**	1.8	0.0	**1.8**	0.5	0.3	**0.8**	0.5	0.5	**1.0**	0.0	0.0	**0.0**	0.2	0.2	**0.3**	0.8	2.3	**3.2**	1.7	2.0	**3.7**
	*T. cati* B6	1.3	0.5	**1.8**	0.4	0.0	**0.4**	0.0	0.0	**0.0**	1.2	0.0	**1.2**	0.3	0.0	**0.3**	0.0	0.0	**0.0**	0.7	0.8	**1.5**	1.5	0.5	**2.0**
	*T. canis* B6	0.0	0.2	**0.2**	12.0	13.6	**25.6**	4.2	4.8	**9.0**	6.3	4.7	**11.0**	4.8	3.2	**8.0**	10.2	6.8	**17.2**	10.8	9.5	**20.3**	5.8	7.3	**13.2**
Cerebellum	*T. cati* Balb/c	0.2	1.4	**1.6**	1.8	1.2	**3.0**	2.7	2.3	**5.0**	1.2	1.2	**2.3**	0.2	0.0	**0.2**	0.2	0.2	**0.3**	3.7	8.3	**12.0**	10.3	7.7	**18.0**
	*T. cati* B6	0.2	0.8	**1.0**	0.8	1.2	**2.0**	2.2	1.2	**3.4**	1.8	3.0	**4.8**	0.3	0.3	**0.5**	0.2	0.2	**0.3**	3.3	2.0	**5.3**	2.8	4.2	**7.0**
	*T. canis* B6	0.0	0.2	**0.2**	3.0	3.6	**6.6**	2.5	5.2	**7.7**	2.0	2.2	**4.2**	1.5	2.0	**3.5**	6.0	4.8	**10.7**	7.5	6.8	**14.3**	4.7	3.5	**8.2**
Total brain	*T. cati* Balb/c	1.6	2.6	**4.2**	3.6	1.2	**4.8**	3.2	2.7	**5.8**	1.7	1.7	**3.3**	0.2	0.0	**0.2**	0.3	0.3	**0.7**	4.5	10.7	**15.2**	12.0	9.7	**21.7**
	*T. cati* B6	1.5	1.3	**2.8**	1.2	1.2	**2.4**	2.2	1.2	**3.4**	3.0	3.0	**6.0**	0.5	0.3	**0.8**	0.2	0.2	**0.3**	4.0	2.8	**6.8**	4.3	4.7	**9.0**
	*T. canis* B6	0.0	0.3	**0.3**	15.0	17.2	**32.2**	6.7	10.0	**16.7**	8.3	6.8	**15.2**	6.3	5.2	**11.5**	16.2	11.2	**27.8**	18.3	16.3	**34.7**	10.5	10.8	**21.3**
Spinal cord	*T. cati* Balb/c	1.0			0.7			0.3			1.0			0.0			0.0			0.0			0.6		
		[0.39]			[0.32]			[0.25]			[0.50]			[0.00]			[0.00]			[0.00]			[0.19]		
	*T. cati* B6	0.9			1.3			0.4			1.1			0.3			0.0			0.0			0.4		
		[0.48]			[2.02]			[0.19]			[0.45]			[0.13]			[0.00]			[0.00]			[0.30]		
	*T. canis* B6	0.0			1.3			0.4			0.9			0.9			0.6			1.1			1.5		
		[0.00]			[3.46]			[0.64]			[1.85]			[1.77]			[0.09]			[2.03]			[3.31]		
Eyes	*T. cati* Balb/c	1.6			0.7			0.0			0.0			0.0			0.0			0.1			0.6		
		[1.01]			[0.63]			[0.00]			[0.00]			[0.00]			[0.00]			[0.07]			[0.31]		
	*T. cati* B6	0.3			0.1			0.0			0.3			0.0			0.0			0.0			0.0		
		[0.32]			[0.65]			[0.00]			[0.15]			[0.00]			[0.00]			[0.00]			[0.00]		
	*T. canis* B6	0.0			0.4			0.3			0.1			0.1			0.3			0.5			0.1		
		[0.00]			[2.84]			[0.4]			[0.42]			[0.12]			[0.32]			[0.70]			[0.23]		

Mean larval numbers as well as recovery rates in [%] for examined eyes, spinal cord and brain (brain recovery rates are pictured in Figures 2 and 3). Brain larval numbers are divided into total brain as well as cerebrum and cerebellum (*LH* = left hemisphere; *RH* = right hemisphere; *T* = total organ). Total numbers may slightly differ as calculations are based on individual mean numbers and therefore, slight discrepancies occasionally occur when totaling values of different brain parts.

### Macroscopical changes and histopathology

Macroscopical observation of removed organs showed severe hemorrhages in lungs on day 2 pi in Balb/c *T. cati* infected mice (4/7 mice, 57.14%) as well as in B6 *T. cati* infected mice (1/8 mice, 12.5%). No lesions were observed in *T. canis* infected mice. On day 7 pi, lungs of all infection groups were affected showing severe hemorrhages (Balb/c *T. cati* infected: 2/7 mice, 28.57%; B6 *T. cati* infected: 4/7 mice, 57.14%; *T. canis* infected: 3/7 mice, 42.86%). Lesions were mainly transient and only observed sporadically after day 14 pi. However, in Balb/c *T. cati* infected mice, hemorrhages in lungs reappeared on day 42 pi (3/8 mice, 37.50%) and were also observed on day 98 pi (4/5 mice, 80.00%).

On day 14 pi, *T. canis* infected mice showed petechial hemorrhages on the liver surface and multifocal retractions of the renal parenchyma (5/8 mice, 62.5%, respectively). Those changes in liver and kidney were not observed in either *T. cati* infection group. Starting day 21 pi until day 42 pi, spleen surfaces infrequently showed focal hyperemia in all infection groups.

**Figure 5:**
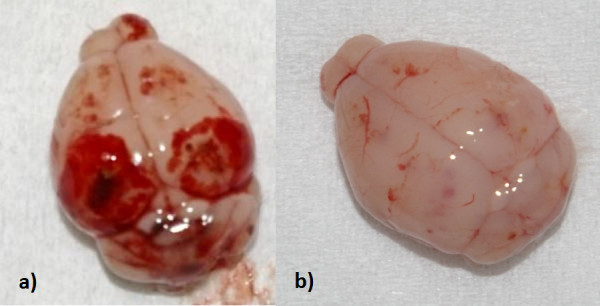
**Macroscopical changes in the brain.** Hemorrhagic lesions in brains of **a)** B6 *T. canis* infected mice and **b)** B6 *T. cati* infected mice on day 7 pi.

**Figure 6:**
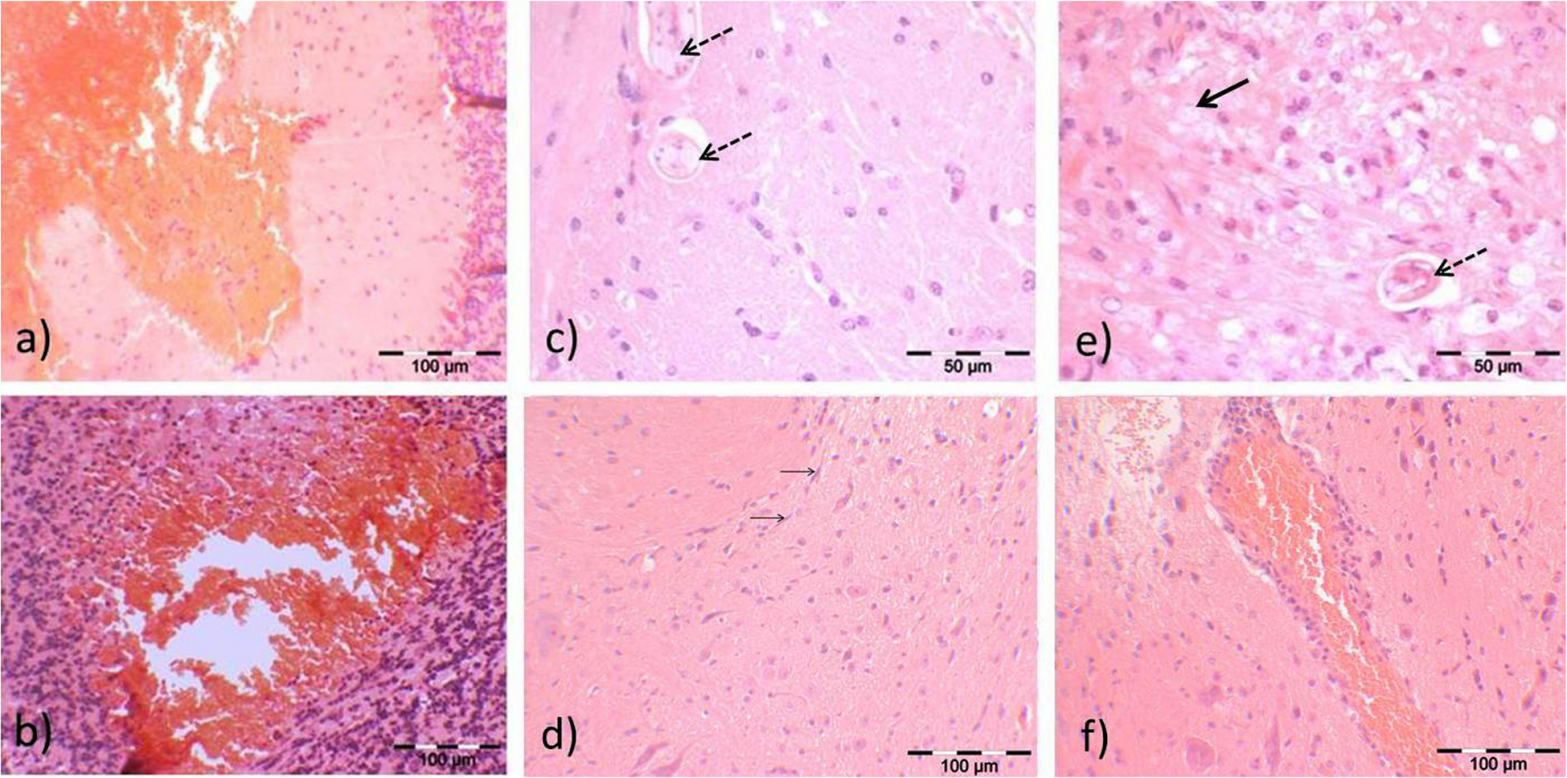
**Histopathological changes in brain structure.** Structural damage observed in brains of experimentally infected B6 mice during the course of infection. **a)***T. canis* and **b)***T. cati* infected mice on day 7 pi. Visible hemorrhages confirm macroscopic observations (cf. Figure 5). **c)***T. canis* and **d)***T. cati* infected mice day 42 pi. In **c)***T. canis* larvae (dashed arrows) are detected in the neuroparenchyma. In **d)**, mild microgliosis is observed. Thin arrows show activated microglia. **e)***T. canis* and **f)***T. cati* infected mice day 98 pi. Structural damage has intensified since day 42 pi. Generally, in *T. canis* infected mice structural damage was evaluated as more severe than in *T. cati* infected mice. In **e)**, arrested or migrating *T. canis* larvae (dashed arrows) can be found. Malacia with demyelination and accumulation of gitter cells (thick arrows) is frequently observed. In **f)**, perivascular lymphocytosis is visualized.

No changes were observed macroscopically or microscopically on day 2 pi in any of the brains. Starting day 7 pi, severe hemorrhages (Figure 5) were observed macroscopically in the brains of both *T. canis* and *T. cati* infected mice (Balb/c *T. cati* infected: 2/7 mice, 28.57%; B6 *T. cati* infected: 1/7 mice, 14.29%; B6 *T. canis* infected: 3/7 mice, 42.86%), which were also seen histopathologically (Figure 6a). Hemorrhages were mainly observed in the brain cortex and were more severe in *T. canis* than *T. cati* infected mice. As in lungs, hemorrhages were reabsorbed starting at day 14 pi and only observed sporadically during the subsequent course of infection. Histopathology revealed changes in brain structure in all infection groups; however, changes in *T. canis* infected mice were more severe than those in *T. cati* infected mice. From day 14 pi onwards, structural brain damage intensified in all infection groups, reaching a maximum of structural damage on the last study day 98 pi. It was apparent that structural damage was mainly observed in the cerebellum of *T. cati* infected mice whereas structural damage was also frequently observed in the cerebrum of *T. canis* infected mice. Histological changes included malacia (*T. canis* infected mice) with the presence of activated microglia and focal accumulation of gitter cells, which are phagocytic cells with the presence of myelin debris within the cytoplasm (myelinophages). Furthermore, occurrence of swollen axons (spheroids), indicative of axonal damage, was observed. Structural damage is shown exemplarily in Figure 6. The subjective impression was that *T. canis* infected mice started to exhibit slight balance problems starting around day 70 pi. Both *T. canis* and *T. cati* infected B6 mice seemed less aggressive day 98 pi when compared to earlier time points.

## Discussion

The migrational route of *T. canis* larvae in the paratenic host, especially in mice, has been examined in various studies resulting in a strong affinity to the brain [10,33-35]. However, very few studies deal with the migrational behaviour of *T. cati* larvae in the paratenic host even though the zoonotic potential is not sufficiently evaluated and should therefore not be underestimated [11]. Direct comparison of studies dealing with either *T. canis* or *T. cati* is difficult and inaccurate as the course of infection is mouse strain dependent as well as depending on infection dose and day post infection [18,28,30,31,36,37]. To overcome this shortcoming, the present study provides a direct comparison of both roundworm species in mice with specific focus on the CNS in order to demonstrate possible brain damage caused by *T. cati* and *T. canis* larvae, respectively, during the acute and the chronic phase of neurotoxocarosis.

Concerning macroscopical changes in the brain, hemorrhagic lesions observed on day 7 pi in the infection groups were also confirmed by histology. It has been proposed that those lesions are caused by larval penetration of arteries in the cortex resulting in injury and consequent bleeding [38]. Lesions within the first week of infection with resulting reabsorption had also been observed in *T. cati* and *T. canis* infected mice by other authors [15,38,39] but could not be observed in a study by Dubey [17]. Histology revealed the presence of malacia in *T. canis* infected mice. Activated microglia and gitter cells, possibly indicating demyelination, as well as spheroid formation as a hallmark of axonal damage were observed in all infection groups. These changes are considered a response of the brain to the migrating or arrested larvae.

The general data obtained show that there are significant differences between infection groups during the acute phase of infection. The higher larval numbers of *T. canis* in the liver in contrast to significantly higher numbers of *T. cati* larvae in the lungs on day 2 pi provides evidence that *T. canis* larvae migrate at a slower pace than *T. cati* larvae. In a previous study, the migrational route was divided into two phases namely the hepato-pulmonary phase and the visceral phase. During the hepato-pulmonary phase, larvae penetrate the intestinal wall after hatching and migrate to the liver. From there on, migration is continued via systemic circulation through which larvae reach the lungs [40]. The slower migration pace of *T. canis* has been observed in mice before [16], however, other studies indicated a slower migration route of *T. cati* in the Mongolian gerbil as paratenic host [33].

The *T. cati* course of infection was unexpected as total recovery rates severely dropped on day 14 pi as well as day 28 and 35 pi in Balb/c *T. cati* infected mice and on day 28 and 35 pi in B6 *T. cati* infected mice. In previous studies, inconsistent recovery rates (between 25%-65%) have been observed; however, those studies solely investigated day 1–28 pi and not the later time points post infection [17]. Initially, it could be assumed that larvae are eliminated from the host’s tissue, however, the increase of recovery rates starting day 42 pi leads to the conclusion that an active migration and shift of location occurs between day 28 and 35 pi as larval numbers in liver and lungs repeatedly increase starting day 35 pi as well as larval numbers in the brain starting day 42 pi. Nevertheless, the tissue-residency and migrational route of larvae during these time points remains unclear. Overall, recovery rates of *T. cati* larvae were up to 16%, which is comparable to about 15.9% on average in gerbils and higher than about 2.0% found in rats [41]. Recovery rates of up to 65.7% of the total infection dose have been determined previously, however, mice received 1000 *T. cati* eggs, i.e. only half of the infection dose of the present study and only 2 mice were investigated per time point post infection leading to a presumably shifted average [15].

By contrast to previous studies on *T. canis* migration in the paratenic host which revealed recovery rates of up to 43.4% in B6 mice after infection with 1000 infective *T. canis* larvae [18,30], comparably low *T. canis* recovery rates of only up to 3.75% were found in the present study. Mechanisms such as trapping of *T. canis* larvae, especially in B6 mice, in the liver had been hypothesized before [42,43]. Possibly, the high amount of infective eggs led to a more sufficient host response and larvae were prevented from entry into the systemic circulation and were simultaneously eliminated. Another explanation is insufficient hatching of eggs and subsequent excretion with the feces [16,17].

Even though low recovery rates were obtained in *T. canis* infected mice, the previously demonstrated strong affinity to the CNS was confirmed in this study. Even though occasionally more than 50% of recovered larvae were detected in muscle tissue, larval numbers may be extrapolated when taking the mass of brain and muscle into consideration resulting in an assumed affinity to brain tissue, which may also be a result of better larval adjustment to brain tissue as opposed to other body tissues. Contrarily, significantly more *T. cati* larvae were found in muscle tissue than in brain tissue during all time points post infection. It has been hypothesized that the pronounced affinity of *T. canis* to the CNS is explained by the size difference when compared to *T. cati* larvae. Consequently, smaller *T. cati* larvae are able to leave arteries more easily, whereas *T. canis* larvae may finally arrest in the brain, as they are not likely to leave the circulatory system [38,42]. However, *T. cati* larvae were recovered in brains of both mouse strains whereas other studies indicated that migration of *T. cati* to the brain occurs rarely or not at all [9,17,18]. The increase of *T. cati* larvae in the brain during later time points post infection was also observed previously, when larval numbers increased starting at day 70 pi [41] as opposed to the current study where larval numbers in the brain start increasing at day 42 pi.

Looking at the left and right hemisphere, significant differences were detected between, but not within the infection groups. The significance of this observation needs to be further investigated as no data concerning left and right hemispheres are available. Additionally, structural damage appears to be consistently distributed in both hemispheres.

The frequent occurrence of *T. cati* larvae in the cerebellum as opposed to *T. canis* larvae in the cerebrum is rather surprising as previous studies demonstrated *T. canis* and *T. cati* larvae being predominantly present in the cerebellum [9,10,33,38]. Eventually, different paratenic hosts could lead to the shift of distribution in the brain. Additionally, due to size difference, the cerebrum is supplied by a greater amount of blood compared to the cerebrum and therefore more *T. canis* larvae may arrest in the small cerebrum arteries. Nevertheless, data obtained correlate well with histopathology as several changes were found in cerebra of *T. canis* infected mice, however, most structural damage in *T. cati* infected mice was observed in the cerebellum. As the cerebellum controls complex motor functions, the predominant occurrence of *T. cati* larvae in the cerebellum may be explained by possible clinical consequences like an increase of immobility and behavioral alterations, e.g. spending more time in open areas. Consequently, paratenic hosts are an easier prey for final hosts of *Toxocara* spp. [26,29,44,45].

Ocular and spinal cord involvement was demonstrated for all infection groups in the present study. Contrarily, previous studies solely demonstrated *T. canis* but not *T. cati* larvae in eyes of infected mice [9,16]. As only very low numbers of larvae were recovered from the eyes, it is apparent that mice are not a suitable model for *T. canis* or *T. cati* induced ocular larva migrans. However, it may not be excluded that OLM and possibly resulting symptoms may occur after infection with either species. Spinal cord involvement has been demonstrated previously in *T. canis* infected mice [9,39] as well as rats, gerbils and hamsters [10], but information about spinal cord involvement in *T. cati* infected mice has not been available so far.

Even though lower percentages of *T. cati* larvae accumulate in the brain, intermittently, comparable numeric values of larvae to *T. canis* were found. Therefore, the risk of neurotoxocarosis caused by *T. cati* should not be underestimated. However, it has also been demonstrated in *T. canis* infected animals that the observed damages in brain structure do not necessarily correspond to the number of larvae present and that a high larval burden does not correlate with the severity of symptoms. Therefore, adverse immune mechanisms are hypothesized to be responsible for the pathology and not the migrating larvae [30]. Additionally, effects of larvae are assumed to be indirect as observed lesions were found to exist separately from the migrating larvae [33]. The low recovery rate of *T. cati* larvae may suggest mild or no brain damage, however, the host’s immune mechanisms may still result in distinct pathology - as it was obvious by histopathological examination of the brains (cf. Figure 6).

The infection risk for humans with *T. cati* has to be considered at least as high as infection risk with *T. canis* as prevalences for example in Europe, range from 3.5%-34% in dogs and from 8%-76% in cats [46-49] whereby cats are more likely to contaminate places like playgrounds, gardens, etc. due to their defecation habits. The resulting environmental contamination increases the risk of infection for humans. Additionally, consuming undercooked or raw meat serves as a transmission route, as several studies have demonstrated the persistence of *T. canis* and *T. cati* larvae in a number of paratenic hosts, which are sources of food for humans [50-53]. Furthermore, viable larvae of *T. canis* as well as *T. cati* have been found in meat even after prolonged periods of freezing [7,8,54,55].

## Conclusions

Even though lower numbers of *T. cati* than *T. canis* larvae were recovered from the brain, the zoonotic potential of *T. cati* should not be underestimated as indirect effects of the larvae may cause distinct pathology. The assumption that most human cases of neurotoxocarosis are caused by *T. canis* should be revised as it was shown, that even present in small numbers, structural damage in *T. cati* infected brains is found. Chronic symptoms after day 98 pi - the last day of the present study - remain to be determined as larval numbers increase starting day 42 pi throughout day 98 pi. Further molecular studies analyzing the host’s response to migrating or persisting larvae in the brain could provide a solid foundation for further investigations on neurotoxocarosis and evaluation of the disease. The occurrence of *T. cati* induced neurotoxocarosis and persistence of infective larvae in meat, even under severe conditions, highlights the need to characterize the course of infection to extrapolate it to human neurotoxocarosis as it may not be as rare as previously thought.

## Competing interests

The authors declare that they have no competing interests.
